# Swahili translation and cultural adaptation of the pediatric patient-reported outcomes version of the common terminology criteria for adverse events (PRO-CTCAE)

**DOI:** 10.1186/s41687-023-00598-4

**Published:** 2023-06-12

**Authors:** Kristin M. Schroeder, Tyler Rizzieri, Ryan R. Lion, Norbert Mtenga, Mwitasrobert Gisiri, Molly McFatrich, Bryce B. Reeve

**Affiliations:** 1 Duke Medicine, Durham, NC USA; 2 Bugando Medical Centre, Mwanza, Tanzania; 3 Stanford Medicine, Stanford, USA; 4 Duke Department of Population Health Sciences, Durham, USA

**Keywords:** PRO-CTCAE, Linguistic validation, Swahili translation, Adverse events, Patient-reported outcome

## Abstract

**Background:**

The pediatric patient-reported outcomes version of the common terminology criteria for adverse event measure was developed and validated for use in pediatric cancer clinical trials to better capture the symptom experiences through direct self-report. The study aim was to develop and validate a Swahili language version of the patient-reported outcomes version of the common terminology criteria for adverse event measure.

**Methods:**

The pediatric version of 15 core symptom adverse events, and the corresponding questions, were selected from the patient-reported outcomes version of the common terminology criteria for adverse event library, then forward and back translated into Swahili by bilingual translators. The translated items were further refined using concurrent cognitive interviewing. Each round of interviews included five children, ages 8–17 years-old, receiving cancer therapy at Bugando Medical Centre, the cancer referral hospital for Northwest Tanzania, and continued until at least 80% of participants understood the question.

**Results:**

Three rounds of cognitive interviews were completed involving 13 patients and 5 caregivers. Among patients, 50% of questions (19/38) were fully comprehended after the first interview round. Two Adverse Events (anxiety and peripheral neuropathy) were the most difficult for participants to understand, associated with education level and experience. Goal comprehension was achieved after three rounds of interviews with no further revisions required. All parents in the first cognitive interview group comprehended the survey, with no additional revisions.

**Conclusion:**

A Swahili patient-reported outcomes version of the common terminology criteria for adverse event was effective in eliciting patient-reported Adverse Events related to cancer treatment, with good comprehension for children aged 8–17 years. This survey is important to incorporate patient self-reporting of symptomatic toxicities and is an effective tool to increase capacity for pediatric cancer clinical trials throughout East Africa, further reducing global disparities in cancer care.

**Supplementary Information:**

The online version contains supplementary material available at 10.1186/s41687-023-00598-4.

## Introduction

Treatment outcomes for children with cancer have improved significantly over the past 60 years, with survival rates of greater than 80% achieved for most pediatric cancer diagnoses in high income countries [1]. These significant survival gains are in large part due to high patient participation in collaborative clinical research trials through large consortiums in North America and Europe [2, 3]. However, over 85% of the 400,000 children diagnosed worldwide live in low- and middle-income countries (LMIC) where survival rates are often below 25% [4]. To address this survival gap and improve patient outcomes, adapted clinical trials have been developed for use in LMICs and successfully implemented in resource constrained regions around the world [5–8].

As part of clinical trial participation, data pertaining to experienced adverse events (AEs) are routinely collected to inform the toxicity profile of the treatment protocol to direct preventative screening and supportive care management. In oncology trials, it is standard practice for clinicians to grade and report all AEs using National Cancer Institute’s (NCI) Common Terminology Criteria for Adverse Events (CTCAE). However, clinician-based evaluation of symptoms has been shown to underreport symptomatic toxicities [9, 10]. Thus, inclusion of patient-reported data may enhance precision and comprehensiveness in the capture of symptomatic adverse effects of cancer treatment. The NCI’s Patient-Reported Outcomes version of the Common Terminology Criteria for Adverse Event library (PRO-CTCAE) was designed for adult patients to self-report symptom AEs they experience while undergoing cancer treatment [11, 12]. There is strong evidence that children may also accurately report their symptomatology experiences.

A group of over 150 clinical experts, PRO methodologists, and patient advocates reviewed CTCAE symptomatic AE terms to determine those that were highly salient for children and adolescents ages 7–17 years undergoing cancer treatment, and used to inform a pediatric version of the PRO-CTCAE (Ped-PRO-CTCAE). The Ped PRO-CTCAE underwent vigorous content and construct validity and has been used in pediatric cancer clinical trials to better capture the symptom experiences through direct self-report by children and adolescents or by caregiver proxy when the child is not able to self-report [6, 7, 11–13]. The final Ped-PRO-CTCAE measurement system includes a library of 130 items (i.e., questions and response options) that can assess up to 62 symptomatic AE concepts. This includes 15 “core” AEs that were found to be relevant across a range of treatments for childhood cancer types. Each symptomatic AE is assessed by 1–3 items representing different symptom attributes (frequency, severity, and interference with activities) with a 4-point ordinal response scale (e.g., “In the past 7 days, how often did you have pain in your mouth or throat—never, sometimes, most of the time, all of the time”). Each individual item is scored separately yielding up to three scores per symptomatic toxicity.

The Ped-PRO-CTCAE was developed for use among English speaking pediatric cancer patients in the United States, and an equivalent translation and adaptation process is required for use in non-English speaking populations to be included in multinational clinical trials. The translation of survey instruments for health research is a complex process involving both the language translation as well as cognitive testing to ensure phrasing is adapted to appropriately convey concepts that are specific to a particular region, country or population. Translation and linguistic validation of the adult PRO-CTCAE have been successfully completed in several languages, and the Ped-PRO-CTCAE has been translated for use among native speakers of Italian and Chinese (Simplified) [14–17, 23].

As Swahili is one of the most common African languages with over 100 million speakers globally, the availability of a Swahili language version of the Ped-PRO-CTCAE is crucial in adding and incorporating patient self-reporting of symptomatic toxicities into cancer clinical trials throughout East Africa and allows for cross study comparisons [18]. The aim of this study was to develop a Swahili language version of the Ped-PRO-CTCAE measure for use in future pediatric cancer clinical trials in East Africa and other Swahili speaking communities. Therefore, comprehension by both children and caregivers was completed to translate the pediatric self-report and parent proxy Ped-PRO-CTCAE, respectively.

## Methods

### Setting

This study was completed at Bugando Medical Centre (BMC), a tertiary, urban hospital located in Mwanza, Tanzania. Cancer services started in 2009 at BMC, and it is one of three comprehensive cancer treatment centers in the country, serving a catchment area of 18 million people in the Lake Zone.

### Subjects

Given that the Ped-PRO-CTCAE can be self-reported or reported by parent proxy, comprehension was evaluated among both patients and caregivers. Over a one-month period, children aged 7–17 years who were being treated for cancer at BMC and their caregivers were invited to participate in this study. Inclusion criteria included the ability to speak and understand Swahili. Proxy consent was obtained from caregivers, with patients over the age of 12 providing assent.

### Translation process and development

**Fig. 1:**
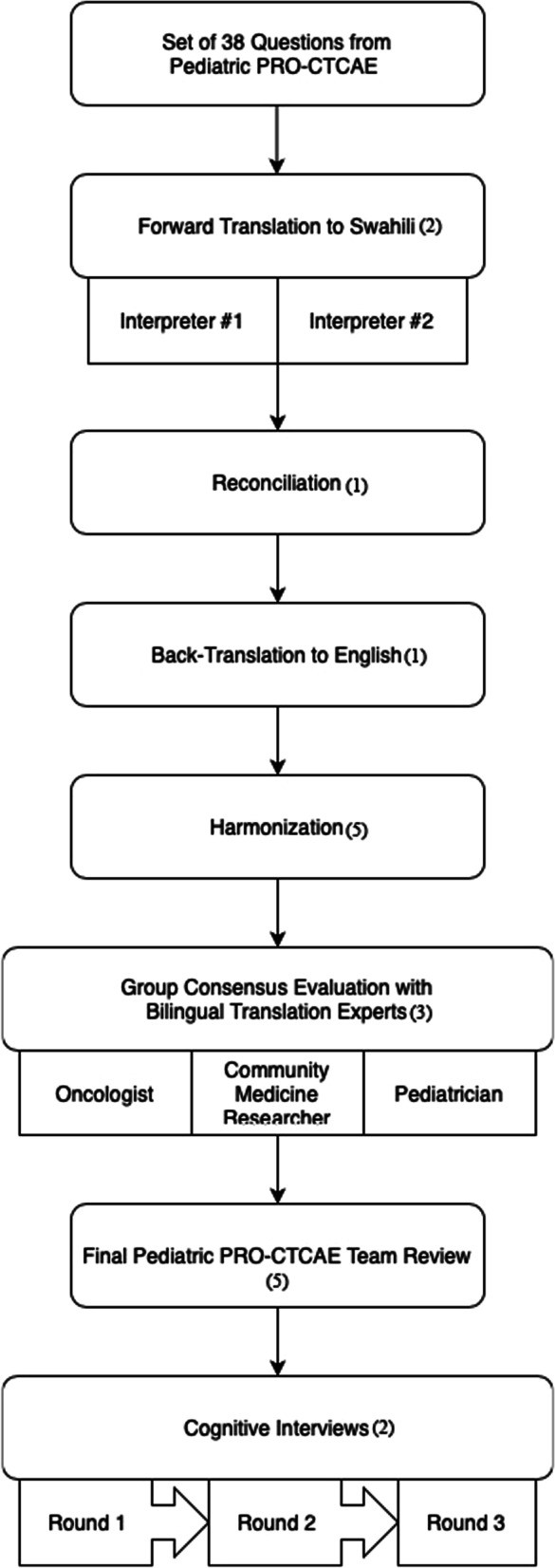
Diagram of the 6 steps used to create the Swahili version of the Questionnaire. “(#)” indicates number of individuals involved in each step

The translation and cultural adaptation was completed per the International Society for Pharmacoeconomics and Outcome Research (ISPOR) guidelines with guidance from the developers of the original Ped-PRO-CTCAE version to ensure that the Swahili version was conceptually equivalent to the original [11, 18]. A team of six native Swahili speakers representing different Swahili speaking regions in both Tanzania and Kenya were assembled. A total of 15 core symptomatic AE concepts, that include 38 questions, were selected from the Ped-PRO-CTCAE library. Each symptomatic AE included in Ped-PRO-CTCAE can include 1–3 questions about the symptom frequency, presence, severity, and interference with daily activities. Figure 1 provides an overview of the translation procedure.

*Step 1 Forward Translation I* The initial translation into Swahili was performed independently by two medical providers that were bilingual native speakers in English and Swahili (JK, BM).

*Step 2 Reconciliation* A third non-medical translator reviewed the original English version along with the two independent Swahili translations and reconciled the differences in the Swahili translation or made a new translation if deemed applicable, generating a single, corrected version of the Swahili questionnaire (HS).

*Step 3 Back-Translation* The reconciled forward translation was then blindly back-translated into English by a fourth non-medical independent translator (HD).

*Step 4 Harmonization* The research team reviewed the original and back translated English versions to identify any errors or potential phrase misuse. These were further reviewed by reconciliation translator (HS) and included into a final reconciled forward translation.

*Step 5 Final Forward Translation* To ensure that there were no conceptual discrepancies between the original English and translated Swahili versions, the final forward translation underwent an independent review by three independent bilingual translation experts, including an oncologist, pediatrician, and community medicine researcher with questionnaire development experience (EA, MG, FB). They reviewed the entire translation history and selected the most appropriate translation for each item or provided an alternate translation if previous translations were not acceptable.

*Step 6 Final Pediatric PRO-CTCAE Team Review* The complete translation process along with the corresponding comments were then reviewed by the Ped-PRO-CTCAE team (KS, MM, BR, TR, MG) in order to develop the finalized version of the Ped-PRO-CTCAE questionnaire used in the cognitive interview phase for linguistic validation.

### Cognitive interviews

To verify that the meaning of each question was properly captured and able to be understood by the intended audience, two interviewers trained in cognitive interviewing methodologies administered the questionnaire to the study participants and their caregivers (TR, NM). The interviews were administered in a select, quiet area at the oncology clinic. Each interview was audio recorded and timed for reference when an unclear response was noted during the interview. With each attempt, the participant was instructed to read and complete the questionnaire by themselves. If the participant was unable to read, the staff read the questionnaire out loud and recorded their responses. During the first round, participants completed the Swahili translated Ped-PRO-CTCAE in its entirety. The interviewers provided the core questionnaire to participants and explored concepts that were difficult to understand using concurrent probing. Any words, phrases and questions that were difficult to understand in the instructions, question stems or response options were recorded for additional review. At the conclusion of each interview, the interviewers wrote down their overall interpretations and recommendations for further question clarification. The recommendations from each of the five interviews were pooled to inform revisions to the translated Ped-PRO-CTCAE questionnaire. When evaluating comprehensibility, more attention was given to Ped-PRO-CTCAE items in which two or more participants had difficulty understanding. If a single person had difficulty understanding a word or a phrase, the interviewers evaluated if it was an isolated comprehension issue or if the translation itself was problematic. If revisions were made to the Swahili translation of the Ped-PRO-CTCAE, an additional round of cognitive interviews were completed until a comprehension goal of at least 80% was reached.

## Results

### Participants’ characteristics

Five caregivers and 13 patient participants were consented to this study. Two children aged 7 were not able to understand the concept and were not enrolled. One participant in the final round of cognitive interviewing was removed by the study team due to perceived discomfort with answering questions on their own. Therefore, in round 3, only 4 participants were included. Two participants reviewed different versions of the questionnaire in 2 separate rounds (rounds 2 and 3). Patient demographics are outlined in Table 1.

**Table 1:** Patient participant demographics in 3 rounds of questionnaires (n = 12)*

Median age (range in years)	13 (8–17)
Age range (number of participants)	8–11 (3)
	12–14 (5)
	15–17 (4)
*Gender (%)*	
Male	6 (50)
Female	6 (50)
*Current education level (%)*	
Primary grade	8 (67)
Secondary grade	4 (33)

*One participant excluded

### Final translation

Fifteen core AEs were selected to be translated from English to Swahili and be regularly tested with the pediatric patients enrolled in clinical oncology trials. The 15 AEs and the corresponding final translation of each symptom after three rounds of revisions are listed in Table 2.

**Table 2:** Final 15 core adverse events translated into Swahili

Symptom in English	Symptom in Swahili
Stomach pain	Maumivu ya tumbo
Problems not being able to poop	Tatizo la kutopata choo/haja kubwa
Runny or watery poop	Umeharisha au haja kubwa ya majimaji
Pain inside your mouth	Maumivu katika mdomo au koo
Sick to your stomach	Kichefuchefu
Throw up	Umetapika
Feeling tired	Kuchoka
General pain	Maumivu
Not want to eat meals	Kutotaka kula chakula
Head hurt	Maumivu ya kichwa
Numbness or tingly feeling in hands or feet (hand or foot falling asleep)	Kuhisi ganzi (kama mkono au mguu kulala)
Worried or nervous	Wasiwasi
Sad or unhappy feelings	Huzuni au kutokuwa na furaha
Problem falling or staying asleep	Tatizo la kutopata usingizi
Cough	Kukohoa

### Patient and proxy comprehension

After the first round of cognitive interviews, all questions were understood by the adult caregivers. However, among the patients, only half of the questions were fully understood by each participant and remained unchanged in the final translation. There were no issues with the instructions, duration, or temporal phrasing within the response options. Six of the evaluated symptoms required at least one round of revisions (Table 3).

**Table 3:** Ped-PRO-CTCAE items presenting comprehension difficulties in rounds 1–3 with subsequent revisions

Symptom wording in English	Symptom wording in Swahili (Round 1)	Participants with difficulty in Round 1 (%)	Revision made	Symptom wording in Swahili (Round 2)	Participants with difficulty in Round 2 (%)	Revision made	Symptom wording in Swahili (Round 3)	Participants with difficulty in Round 3 (%)
General Pain	Maumivu	2/5 (40)	Moved to different question order in the survey	Maumivu	0/5 (0)	N/A	N/A	N/A
Problems with not being able to poop	Tatizo la kutopata haja kubwa	2/5 (40)	Added alternative word meaning “defecate” to probe and compare if “poop” or “defecate” was more comprehensive	Tatizo la kutopata choo/haja kubwa	1/5 (20)	N/A	N/A	N/A
Pain in your mouth or throat	Maumivu katika kinywa chako au koo	2/5 (40)	Change word “in” to “inside” your mouth or throat	Maumivu katika mdomo au koo	0/5 (20)	N/A	N/A	N/A
Numbness or tingly feeling in your hands or feet	Kuhisi ganzi	2/5 (40)	Add example “the feeling you get when you sleep on your arm or leg”	Kuhisi ganzi (Inafanana na ile hali unayopata baada ya kulalia mkono au kukalia mguu moja vibaya)	2/5 (40)	Add example “hand or foot falling asleep” to the question	Kuhisi ganzi (kama mkono au mguu kulala)	1/4 (25)
Worried or nervous	Woga au hofu	3/5 (60)	Add example of “feeling you get before having to take a test”	Woga au hofu	2/5 (40)	Removed example and changed word for worried to “wasi wasi”	Wasiwasi	0/4 (0)
Problems Sleeping	Tatizo la kupata usingiz	3/5 (60)	Add “or” in the question stem: Trouble falling asleep or waking up	Tatizo la kupata usingiz	2/5 (40)	Word change from “kupata” to “kutopata”	Tatizo la kutopata usingizi	0/4 (0)

*N/A = item not recommended for further revision, not tested in round 3; one patient in round three was excluded from the study

### Final recommendations

Overall, all but one question obtained 100% agreement after three rounds of cognitive interviews and revision. Questions on peripheral neuropathy demonstrated difficulty in comprehension depending on prior experience with the respective symptoms. The final version of the questionnaire for the patient (Additional file 1: Appendix 1) and proxy (Additional file 2: Appendix 2) is believed to be the most effective form for patient’s aged 8–18 years old to understand with no further recommendations.

## Discussion

The purpose of this study was to translate a Swahili language version of the Ped-PRO-CTCAE measure, allowing the incorporation of patient self-reporting of symptomatic toxicities for future pediatric cancer clinical trials throughout East Africa. The rigorous translation process ensured that the Swahili Ped-PRO-CTCAE measure had high comprehension and effectiveness in eliciting patient-reported AEs related to cancer treatment.

The key strength of this study is that the methods used to translate and linguistically validate the Swahili Ped-PRO-CTCAE followed the Principles of Good Translation and Cultural Adaptation Practice as articulated by ISPOR. Our approach including initial interviews, forward and backward translations, group consensus evaluation, and three rounds of cognitive testing ensured development of a set of questions with strong clarity and content validity. This methodology has been used to similarly translate and culturally adapt the PRO-CTCAE into languages such as Spanish, German, Dutch, and Italian as well as being used to translate the Ped-PRO-CTCAE into the Chinese and Italian languages, with numerous other translations currently in development [20–22].

During the cognitive interviews, all caregivers understood the presented symptom concepts and only one cognitive interview round was required. However, there were 3 concepts that were challenging for pediatric patients—peripheral neuropathy, anxiety, and difficulty sleeping. Patients who completed the Italian and Chinese translation cognitive interviews understood these concepts, but had more difficulty with dry eyes and throat [17, 23]. Swahili is a Bantu language, influenced by Arabic and to a lesser extent, European language. While straightforward concepts, such as dry eyes, are commonly experienced and therefore have a Swahili phrase, more complex medical terms often do not have a direct translation, and therefore requires descriptive language to describe to concept. However, with some concepts like peripheral neuropathy, we found that even with descriptive language, the phrase was not understood by all participants but was universally understood by those who had experienced the symptom. As such, the Ped-PRO-CTCAE is still likely to successfully elicit the specific symptom toxicities in this population.

The current study was limited to the 15 core AEs that occur across a broad spectrum of pediatric cancer treatments, as was translated in the Italian Ped PRO-CTAE. These were intentionally selected as the most clinically relevant, with the highest correlation to current treatment toxicity monitoring. However, the full panel of 62 AEs can be tested in future studies to allow for more nuanced toxicity evaluation for new drug development and clinical trial testing in Swahili speaking regions. Additionally, this survey was translated using Swahili in a Tanzanian context. While Swahili is spoken throughout East Africa, there are regional linguistic variations and additional language modification may be required if employing the survey in other Swahili speaking populations. Planned future studies will evaluate construct validity and test–retest reliability of the Swahili Ped-PRO-CTCAE survey versus standard clinician evaluation using the CTCAE and symptom assessment scales through prospective enrollment of patient-parent-clinician triads at BMC.

## Conclusion

With low- and middle-income countries (LMICs) accounting for over 85% of the 400,000 newly diagnosed pediatric cancer cases, and the largest gain in new patients seen in Sub Saharan Africa, the Swahili translation provides a critical tool to assess new treatment protocols targeting the almost 60% survival gap reported in this region [1]. This is the first study to translate the Ped-PRO-CTCAE to a Swahili, the most common African language, and spoken by over 100 million people worldwide. Validated patient-reported measures will be essential in the development and evaluation of future clinical trials to reduce pediatric cancer morbidity and mortality worldwide.

## Supplementary Information

**Additional file 1**. Ped-PRO-CTCAE-Survey.

**Additional file 2**. Ped-PRO-CTCAE-Proxy.

## Data Availability

The datasets supporting the conclusions of this article are included within the article and Appendix 1.

## Abbreviations

AE: Adverse event
NCI: National cancer institute
LMIC: Low- and middle-income countries
CTCAE: Common terminology criteria for adverse events
PRO-CTCAE: Patient-Reported outcomes version of the common terminology criteria for adverse event library
Ped-PRO-CTCAE: Pediatric patient-reported outcomes version of the common terminology criteria for adverse event library
BMC: Bugando medical centre
ISPOR: International society for pharmacoeconomics and outcome research
