# Supplementary material for: Swahili translation and cultural adaptation of the pediatric patient-reported outcomes version of the common terminology criteria for adverse events (PRO-CTCAE)
Source: J Patient Rep Outcomes. 2023 Jun 12;7:56. doi: 10.1186/s41687-023-00598-4 (PMC10260717; doi:10.1186/s41687-023-00598-4)
Supplement: Supplementary file 1 — Additional file 1. Ped-PRO-CTCAE-Survey. [file 41687_2023_598_MOESM1_ESM.docx]

Tafadhali jibu kila swali kwa kuonyesha jibu moja kwa kila swali. Tafadhali jibu kila swali kwa kuweka alama katika jibu moja kisanduku kwa kila mstari. Tafadhali fikiria juu ya siku saba zilizopita na ujibu maswali yafuatayo hapa chini.

1. Ndani ya siku saba zilizopita ni mara ngapi umekuwa na maumivu?

- Sikuwa na maumivu kabisa
- Mara chache
- Mara nyingi
- Wakati wote

❑ Swali hili ni gumu kuelewa

1. Ndani ya siku saba zilizopita maumivu yako yalikuwa mabaya kiasi gani?

- Sikuwa na maumivu
- Mabaya kidogo
- Mbaya
- Mabaya sana

❑ Swali hili ni gumu kuelewa

1. Ndani ya siku saba zilizopita, ni kwa kiasi gani maumivu yamekuzuia kufanya shughuli zako ulizozizoea?

- hapana kabisa
- kidogo
- sana
- kwa kiasi kikubwa sana

❑ Swali hili ni gumu kuelewa

1. Ndani ya siku saba zilizopita, ni mara ngapi ulipata maumivu ya tumbo?

- Sikupata kabisa
- Mara chache
- Mara nyingi
- Wakati wote

❑ Swali hili ni gumu kuelewa

1. Ndani ya siku saba ziliozopita ni kwa jinsi gani maumivu ya tumbo lako yamekuwa makali?

- Sikuwa na maumivu
- Makali kidogo
- Makali
- Makali sana

❑ Swali hili ni gumu kuelewa

1. Ndani ya siku saba zilizopita, ni mara ngapi maumivu ya tumbo yalikuzuia kufanya shughuli zako za kawada?

- haijatokea
- mara chache
- mara kwa mara
- wakati wote

❑ Swali hili ni gumu kuelewa

1. Ndani ya siku saba zilizopita, ni mara ngapi ulipata tatizo la kutopata choo/haja kubwa?

- Sikupata
- Mara chache
- Mara nyingi
- Wakati wote

❑ Swali hili ni gumu kuelewa

Ndani ya siku saba zilizopita, ni kwa kiasi gani matatizo ya kutopata choo/haja kubwa yalizidi??

- Sikuwa na tatizo
- Yalizidi kidogo
- Yalizidi
- Yalizidi sana

❑ Swali hili ni gumu kuelewa

1. Ndani ya siku saba zilizopita, ni kwa kiasi gani matatizo yako ya kutopata haja kubwa yamekuzuia kufanya shughuli zako ulizozizoea?

- hapana kabisa
- kidogo
- sana
- kwa kiasi kikubwa sana

❑ Swali hili ni gumu kuelewa

1. Ndani ya siku saba zilizopita,ni mara ngapi umeharisha au kupata haja kubwa ya majimaji?

- Sikuharisha kabisa
- Mara chache
- Mara nyingi
- Wakati wote

❑ Swali hili ni gumu kuelewa

1. Ndani ya siku saba zilizopita ni kwa kiasi gani kuharisha au kupata haja kubwa ya maji maji kumekuzuia kufanya shughuli zako ulizozizoea?

- hapana kabisa
- kidogo
- sana
- kwa kiasi kikubwa sana

❑ Swali hili ni gumu kuelewa

1. Ndani ya siku saba zilizopita,ni mara ngapi umekuwa na maumivu ndani ya mdomo au koo?

- Sikuwa na maumivu
- Mara chache
- Mara nyingi
- Wakati wote

❑ Swali hili ni gumu kuelewa

1. Ndani ya siku saba zilizopita,ni kwa kiasi gani maumivu ndani ya mdomo au koo lako yalikuwa mabaya?

- Sikuwa na maumivu
- Mabaya kidogo
- Mbaya
- Mabaya sana

❑ Swali hili ni gumu kuelewa

1. Ndani ya siku saba zilizopita, ni kwa kiasi gani maumivu ndani ya mdomo au koo lako yamekuzuia kufanya shughuli zako ulizozizoea?

- hapana kabisa
- kidogo
- sana
- kwa kiasi kikubwa sana

❑ Swali hili ni gumu kuelewa

1. Ndani ya siku saba zilizopita, ni mara ngapi ulihisi kichefuchefu?

- Sikuwa na kichefuchefu kabisa
- Mara chache
- Mara nyingi
- Wakati wote

❑ Swali hili ni gumu kuelewa

1. Ndani ya siku saba zilizopita kichefuchefu kilikuwa kibaya kiasi gani?

- Sikuwa na kichefuchefu
- Kibaya kidogo
- Mbaya
- Kibaya sana

❑ Swali hili ni gumu kuelewa

1. Ndani ya siku saba zilizopita ni kwa kiasi gani kichefuchefu kilikuzuia kufanya shughuli zako ulizozizoea?

- hapana kabisa
- kidogo
- sana
- kwa kiasi kikubwa sana

❑ Swali hili ni gumu kuelewa

1. Ndani ya siku saba zilizopita ni mara ngapi umetapika?

- Sikutapika
- Mara chache
- Mara nyingi
- Wakati wote

❑ Swali hili ni gumu kuelewa

1. Ndani ya siku saba zilizopita, ni kwa kiasi gani kutapika kumekuzuia kufanya shughuli zako ulizozizoea?

- hapana kabisa
- kidogo
- sana
- kwa kiasi kikubwa sana

❑ Swali hili ni gumu kuelewa

1. Ndani ya siku saba zilizopita, ni mara umejisikia kuchoka?

- Sikujiskia kabisa
- Mbaya kidogo
- Mbaya
- Mbaya sana

❑ Swali hili ni gumu kuelewa

1. Ndani ya siku saba zilizopita, ni kwa kiasi gani kuchoka kumekuzuia kufanya shughuli zako ulizozizoea?

- hapana kabisa
- kidogo
- sana
- kwa kiasi kikubwa sana

❑ Swali hili ni gumu kuelewa

1. Ndani ya siku saba zilizopita ni mara ngapi hukutaka kula chakula chako?

- Kamwe
- Mara chache
- Mara nyingi
- Wakati wote

❑ Swali hili ni gumu kuelewa

1. Ndani y a siku saba ni mara ngapi umepata maumivu ya kichwa?

- Sikupata kabisa
- Mara chache
- Mara nyingi
- Wakati wote

❑ Swali hili ni gumu kuelewa

1. Ndani ya siku saba zilizopita maumivu ya kichwa yamekuwa makali kiasi gani kwako?

- Sikuwa na maumivu
- Makali kidogo
- Makali
- Makali sana

❑ Swali hili ni gumu kuelewa

1. Ndani ya siku saba zilizopita ni kwa kiasi gani maumivu ya kichwa yamekuzuia kufanya shughuli zako ulizozizoea?

- hapana kabisa
- kidogo
- sana
- kwa kiasi kikubwa sana

❑ Swali hili ni gumu kuelewa

1. Ndani ya siku saba zilizopita hali ya kuhisi ganzi imekuwa mbaya kwako kwa kiasi gani katika miguu/mikono? (kama mkono au mguu kulala)

- Sikuhisi ganzi kabisa
- Mbaya kidogo
- Mbaya
- Mbaya sana

❑ Swali hili ni gumu kuelewa

Ndani ya siku saba zilizopita hali ya kuhisi ganzi katika miguu/mikono kumekuzuia kufanya shughuli zako ulizozizoea kwa kiasi gani?

- hapana kabisa
- kidogo
- sana
- kwa kiasi kikubwa sana

❑ Swali hili ni gumu kuelewa

1. Ndani ya siku saba zilizopita,ni mara ngapi umekuwa na wasiwasi?

- Sikuwa na hofu kabisa
- Mara chache
- Mara nyingi
- Wakati wote

❑ Swali hili ni gumu kuelewa

1. Ndani ya siku saba zilizopita, hali ya wewe kuwa na wasiwasi imekuwa mbaya kiasi gani?

- Sikuwa na woga au hofu
- Mbaya kidogo
- Mbaya
- Mbaya sana

❑ Swali hili ni gumu kuelewa

1. Ndani ya siku saba zilizopita, hali ya wewe kuwa na wasiwasi imekuzuia kufanya shughuli zako ulizozizoea kwa kiasi gani?

- hapana kabisa
- kidogo
- sana
- kwa kiasi kikubwa sana

❑ Swali hili ni gumu kuelewa

1. Ndani ya siku saba zilizopita,ni kwa kiasi gani hali ya huzuni au kutokuwa na furaha imekuwa mbaya kwako?

- Sikuwa na huzuni
- Mbaya kidogo
- Mbaya
- Mbaya sana

❑ Swali hili ni gumu kuelewa

1. Ndani ya siku saba zilizopita,ni kwa kiasi gani hali ya huzuni au kutokuwa na furaha imekuzuia kufanya shughuli zako ulizozizoea?

- hapana kabisa
- kidogo
- sana
- kwa kiasi kikubwa sana

❑ Swali hili ni gumu kuelewa

Ndani ya siku saba zilizopita ni mara ngapi umepata matatizo ya kutopata usingizi au kukaa usingizini bila kushtuka usiku?

- Sikushindwa
- Mara chache
- Mara nyingi
- Wakati wote

❑ Swali hili ni gumu kuelewa

1. Ndani ya siku saba zilizopita hali ya tatizo la kutopata usingizi au kukaa usingizini bila kushtuka usiku ilikuwa mbaya kiasi gani?

- Sikuwa na tatizo
- Mbaya kidogo
- Mbaya
- Mbaya sana

❑ Swali hili ni gumu kuelewa

1. Ndani ya siku saba zilizopita hali ya tatizo la kutopata usingizi au kukaa usingizini bila kushtuka usiku imekuzuia kufanya shughuli zako ulizozizoea kwa kiasi gani?

- hapana kabisa
- kidogo
- sana
- kwa kiasi kikubwa sana

❑ Swali hili ni gumu kuelewa

1. Ndani ya siku saba zilizopita, ni mara ngapi umekohoa?

- Sijakohoa kabisa
- Mara chache
- Mara nyingi
- Wakati wote

❑ Swali hili ni gumu kuelewa

1. Ndani ya siku saba zilizopita,hali ya kukohoa ilikuwa mbaya kiasi gani?

- Sikuwa na kikohozi
- Mbaya kidogo
- Mbaya
- Mbaya sana

❑ Swali hili ni gumu kuelewa

Ndani ya

1. Siku saba zilizopita,hali ya kukohoa imekuzuia kwa kiasi gani kufanya shughuli zako ulizozizoea?

- hapana kabisa
- kidogo
- sana
- kwa kiasi kikubwa sana

❑ Swali hili ni gumu kuelewa
