# Supplementary material for: Swahili translation and cultural adaptation of the pediatric patient-reported outcomes version of the common terminology criteria for adverse events (PRO-CTCAE)
Source: J Patient Rep Outcomes. 2023 Jun 12;7:56. doi: 10.1186/s41687-023-00598-4 (PMC10260717; doi:10.1186/s41687-023-00598-4)
Supplement: Supplementary file 2 — Additional file 2. Ped-PRO-CTCAE-Proxy. [file 41687_2023_598_MOESM2_ESM.docx]

Tafadhali jibu kila swali kwa kuonyesha jibu moja kwa kila swali. Tafadhali jibu kila swali kwa kuweka alama katika jibu moja kisanduku kwa kila mstari. Tafadhali fikiria juu ya siku saba zilizopita na ujibu maswali yafuatayo hapa chini.

1. Ndani ya siku saba zilizopita ni mara ngapi mwanao amekuwa na maumivu?

- Hakuwa na maumivu kabisa
- Mara chache
- Mara nyingi
- Wakati wote

❑ Swali hili ni gumu kuelewa

1. Ndani ya siku saba zilizopita maumivu yake yalikuwa mabaya kiasi gani?

- Hakuwa na maumivu
- Mabaya kidogo
- Mbaya
- Mabaya sana

❑ Swali hili ni gumu kuelewa

1. Ndani ya siku saba zilizopita, ni kwa kiasi gani maumivu yamemzuia kufanya shughuli zako alizozizoea?

- hapana kabisa
- kidogo
- sana
- kwa kiasi kikubwa sana

❑ Swali hili ni gumu kuelewa

1. Ndani ya siku saba zilizopita, ni mara ngapi mwanao alipata maumivu ya tumbo?

- Hakupata kabisa
- Mara chache
- Mara nyingi
- Wakati wote

❑ Swali hili ni gumu kuelewa

1. Ndani ya siku saba ziliozopita ni kwa jinsi gani maumivu ya tumbo lake yamekuwa makali?

- Hakuwa na maumivu
- Makali kidogo
- Makali
- Makali sana

❑ Swali hili ni gumu kuelewa

1. Ndani ya siku saba zilizopita, ni mara ngapi maumivu ya tumbo yalimzuia kufanya shughuli zake za kawada?

- haijatokea
- mara chache
- mara kwa mara
- wakati wote

❑ Swali hili ni gumu kuelewa

1. Ndani ya siku saba zilizopita, ni mara ngapi mwanao alipata tatizo la kutopata choo/haja kubwa?

- Hakupata
- Mara chache
- Mara nyingi
- Wakati wote

❑ Swali hili ni gumu kuelewa

Ndani ya siku saba zilizopita, ni kwa kiasi gani matatizo yake ya kutopata choo/haja kubwa yalizidi??

- Sikuwa na tatizo
- Yalizidi kidogo
- Yalizidi
- Yalizidi sana

❑ Swali hili ni gumu kuelewa

1. Ndani ya siku saba zilizopita, ni kwa kiasi gani matatizo yake ya kutopata haja kubwa yamemzuia kufanya shughuli zake ulizozizoea?

- hapana kabisa
- kidogo
- sana
- kwa kiasi kikubwa sana

❑ Swali hili ni gumu kuelewa

1. Ndani ya siku saba zilizopita,ni mara ngapi mwanao ameharisha au kupata haja kubwa ya majimaji?

- Sikuharisha kabisa
- Mara chache
- Mara nyingi
- Wakati wote

❑ Swali hili ni gumu kuelewa

1. Ndani ya siku saba zilizopita ni kwa kiasi gani kuharisha au kupata haja kubwa ya maji maji kumemzuia kufanya shughuli zake alizozizoea?

- hapana kabisa
- kidogo
- sana
- kwa kiasi kikubwa sana

❑ Swali hili ni gumu kuelewa

1. Ndani ya siku saba zilizopita,ni mara ngapi mwanao amekuwa na maumivu ndani ya mdomo au koo?

- Sikuwa na maumivu
- Mara chache
- Mara nyingi
- Wakati wote

❑ Swali hili ni gumu kuelewa

1. Ndani ya siku saba zilizopita,ni kwa kiasi gani maumivu ndani ya mdomo au koo lake yalikuwa mabaya?

- Sikuwa na maumivu
- Mabaya kidogo
- Mbaya
- Mabaya sana

❑ Swali hili ni gumu kuelewa

1. Ndani ya siku saba zilizopita, ni kwa kiasi gani maumivu ndani ya mdomo au koo lake yamemzuia kufanya shughuli zake alizozizoea?

- hapana kabisa
- kidogo
- sana
- kwa kiasi kikubwa sana

❑ Swali hili ni gumu kuelewa

1. Ndani ya siku saba zilizopita, ni mara ngapi mwanao alihisi kichefuchefu?

- Sikuwa na kichefuchefu kabisa
- Mara chache
- Mara nyingi
- Wakati wote

❑ Swali hili ni gumu kuelewa

1. Ndani ya siku saba zilizopita kichefuchefu kwake kilikuwa kibaya kiasi gani?

- Sikuwa na kichefuchefu
- Kibaya kidogo
- Mbaya
- Kibaya sana

❑ Swali hili ni gumu kuelewa

1. Ndani ya siku saba zilizopita ni kwa kiasi gani kichefuchefu kilimzuia kufanya shughuli zake alizozizoea?

- hapana kabisa
- kidogo
- sana
- kwa kiasi kikubwa sana

❑ Swali hili ni gumu kuelewa

1. Ndani ya siku saba zilizopita ni mara ngapi mwanao ametapika?

- Hakutapika
- Mara chache
- Mara nyingi
- Wakati wote

❑ Swali hili ni gumu kuelewa

1. Ndani ya siku saba zilizopita, ni kwa kiasi gani kutapika kumemzuia kufanya shughuli zake alizozizoea?

- hapana kabisa
- kidogo
- sana
- kwa kiasi kikubwa sana

❑ Swali hili ni gumu kuelewa

1. Ndani ya siku saba zilizopita, ni mara mwanao amejisikia kuchoka?

- Hakujiskia kabisa
- Mbaya kidogo
- Mbaya
- Mbaya sana

❑ Swali hili ni gumu kuelewa

1. Ndani ya siku saba zilizopita, ni kwa kiasi gani kuchoka kumemzuia kufanya shughuli zake alizozizoea?

- hapana kabisa
- kidogo
- sana
- kwa kiasi kikubwa sana

❑ Swali hili ni gumu kuelewa

1. Ndani ya siku saba zilizopita ni mara ngapi mwanao hakutaka kula chakula chake?

- Kamwe
- Mara chache
- Mara nyingi
- Wakati wote

❑ Swali hili ni gumu kuelewa

1. Ndani y a siku saba ni mara ngapi mwanao amepata maumivu ya kichwa?

- Sikupata kabisa
- Mara chache
- Mara nyingi
- Wakati wote

❑ Swali hili ni gumu kuelewa

1. Ndani ya siku saba zilizopita maumivu ya kichwa yamekuwa makali kiasi gani kwake?

- Sikuwa na maumivu
- Makali kidogo
- Makali
- Makali sana

❑ Swali hili ni gumu kuelewa

1. Ndani ya siku saba zilizopita ni kwa kiasi gani maumivu ya kichwa yamemzuia kufanya shughuli zake alizozizoea?

- hapana kabisa
- kidogo
- sana
- kwa kiasi kikubwa sana

❑ Swali hili ni gumu kuelewa

1. Ndani ya siku saba zilizopita hali ya kuhisi ganzi kwa mwanao imekuwa mbaya kwake kwa kiasi gani katika miguu/mikono? (kama mkono au mguu kulala)

- Hakuhisi ganzi kabisa
- Mbaya kidogo
- Mbaya
- Mbaya sana

❑ Swali hili ni gumu kuelewa

Ndani ya siku saba zilizopita hali ya kuhisi ganzi katika miguu/mikono imemzuia kufanya shughuli zake alizozizoea kwa kiasi gani?

- hapana kabisa
- kidogo
- sana
- kwa kiasi kikubwa sana

❑ Swali hili ni gumu kuelewa

1. Ndani ya siku saba zilizopita, ni mara ngapi mwanao amekuwa na wasiwasi?

- Sikuwa na hofu kabisa
- Mara chache
- Mara nyingi
- Wakati wote

❑ Swali hili ni gumu kuelewa

1. Ndani ya siku saba zilizopita, hali ya mwanao kuwa na wasiwasi imekuwa mbaya kiasi gani?

- Sikuwa na woga au hofu
- Mbaya kidogo
- Mbaya
- Mbaya sana

❑ Swali hili ni gumu kuelewa

1. Ndani ya siku saba zilizopita, hali ya mwanao kuwa na wasiwasi imemzuia kufanya shughuli zake alizozizoea kwa kiasi gani?

- hapana kabisa
- kidogo
- sana
- kwa kiasi kikubwa sana

❑ Swali hili ni gumu kuelewa

1. Ndani ya siku saba zilizopita,ni kwa kiasi gani hali ya huzuni au kutokuwa na furaha kwa mwanao imekuwa mbaya kwake?

- Sikuwa na huzuni
- Mbaya kidogo
- Mbaya
- Mbaya sana

❑ Swali hili ni gumu kuelewa

1. Ndani ya siku saba zilizopita,ni kwa kiasi gani hali ya huzuni au kutokuwa na furaha imemzuia kufanya shughuli zake alizozizoea?

- hapana kabisa
- kidogo
- sana
- kwa kiasi kikubwa sana

❑ Swali hili ni gumu kuelewa

Ndani ya siku saba zilizopita ni mara ngapi mwanao amepata matatizo ya kutopata usingizi au kukaa usingizini bila kushtuka usiku?

- Sikushindwa
- Mara chache
- Mara nyingi
- Wakati wote

❑ Swali hili ni gumu kuelewa

1. Ndani ya siku saba zilizopita hali ya tatizo la kutopata usingizi au kukaa usingizini bila kushtuka usiku kwa mwanao ilikuwa mbaya kiasi gani?

- Sikuwa na tatizo
- Mbaya kidogo
- Mbaya
- Mbaya sana

❑ Swali hili ni gumu kuelewa

1. Ndani ya siku saba zilizopita hali ya tatizo la kutopata usingizi au kukaa usingizini bila kushtuka usiku imemzuia kufanya shughuli zake alizozizoea kwa kiasi gani?

- hapana kabisa
- kidogo
- sana
- kwa kiasi kikubwa sana

❑ Swali hili ni gumu kuelewa

1. Ndani ya siku saba zilizopita, ni mara ngapi mwanao amekohoa?

- Hajakohoa kabisa
- Mara chache
- Mara nyingi
- Wakati wote

❑ Swali hili ni gumu kuelewa

1. Ndani ya siku saba zilizopita,hali ya kukohoa kwa mwanao ilikuwa mbaya kiasi gani?

- Sikuwa na kikohozi
- Mbaya kidogo
- Mbaya
- Mbaya sana

❑ Swali hili ni gumu kuelewa

Ndani ya

1. Siku saba zilizopita,hali ya kukohoa imemzuia kwa kiasi gani kufanya shughuli zake alizozizoea?

- hapana kabisa
- kidogo
- sana
- kwa kiasi kikubwa sana

❑ Swali hili ni gumu kuelewa
